# Human adenoviruses in paediatric patients with respiratory tract infections in Beijing, China

**DOI:** 10.1186/s12985-021-01661-6

**Published:** 2021-09-23

**Authors:** Yiman Huang, Chao Wang, Fenlian Ma, Qiong Guo, Lihong Yao, Aijun Chen, Xiaoyi Luo, Lishu Zheng

**Affiliations:** 1 NHC Key Laboratory of Medical Virology and Viral Diseases, National Institute for Viral Disease Control and Prevention, China CDC, Beijing, China; 2 Center for Biosafety Mega-Science, Chinese Academy of Sciences, Beijing, China

**Keywords:** Human adenovirus, Respiratory tract infection, Epidemiology, Genetic diversity

## Abstract

**Background:**

Human adenoviruse (HAdV) is a major pathogen of paediatric respiratory tract infections (RTIs). Mutation or recombination of HAdV genes may cause changes in its pathogenicity and transmission. We described the epidemiology and genotypic diversity of HAdV in hospitalized children with RTIs in Beijing, China.

**Methods:**

Nasopharyngeal aspirates were collected from hospitalized children with RTIs from April 2018 to March 2019. HAdVs were detected by a quantitative real-time PCR, and the hexon gene was used for phylogenetic analysis.

**Results:**

Among 1572 samples, 90 (5.72%) were HAdV-positive. The HAdV detection rate was highest in November and July. Among HAdV-positive children, 61.11% (55/90) were co-infected with other respiratory viruses, the most common of which were human respiratory syncytial virus and human rhinovirus. The main diagnosis was bronchopneumonia, most patient have cough and fever. Children with a high viral load were more likely to have a high fever (*P* = 0.041) and elevated WBC count (*P* = 0.000). Of 55 HAdV-positive specimens, HAdV-B (63.64%), HAdV-C (27.27%), and HAdV-E (9.09%) were main epidemic species. Phylogenetic analysis indicated that hexon sequences of three samples were on the same branch with the recombinant HAdV strain (CBJ113), which was circulating in Beijing since 2016.

**Conclusion:**

The HAdV-B3 and HAdV-B7 are the main epidemic strains in Beijing, and the recombinant HAdV-C strain CBJ113 has formed an epidemic trend.

**Supplementary Information:**

The online version contains supplementary material available at 10.1186/s12985-021-01661-6.

## Background

Both well-known and emerging viruses affect human health by causing various diseases, and sometimes they even have a devastating impact on the entire society, such as the newly emerged human coronavirus, severe acute respiratory syndrome coronavirus 2 (SARS-CoV-2). Adenoviruses (AdVs), members of the family *Adenoviridae*, are non-enveloped double-stranded DNA viruses, found widely in the biosphere. Since they were first discovered by Rowe et al. in 1953 [1], AdVs have been the focus of intense research. AdVs can infect various tissues and organs, sometimes with serious consequences, especially in children. The infectivity and cell entry mechanism of AdVs make them suitable for drug delivery, vaccination and gene therapy for many diseases including cancer [2]. Research on adenoviruses has greatly contributed to the fields of life sciences and medicine over the past decades.

Currently, about 110 human adenovirus (HAdV) genotypes are recognized; these are classified into seven species (A–G) in the genus *Mastadenovirus* based on their physical, chemical and biological properties [3]. HAdV direct or indirect transmission of occurs through throat, faeces, eyes or urine, depending on the virus type. Certain HAdV types are predominantly associated with specific pathologies, such as acute respiratory outbreaks (HAdV-B/C/E) [4, 5], epidemic keratoconjunctivitis (HAdV-D) [6, 7], gastroenteritis, and/or acute hemorrhagic cystitis (HAdV-F/G) [8, 9]. The major disease-associated HAdV genotypes detected in various countries and regions differ and change over time [10]. Although most HAdVs cause only mild symptoms, some can cause severe infections, most of which occur in children, the elderly, and people with severely compromised immune systems [11–13]. However, there have also been reported cases of severe pneumonia and death in adults with normal immunity caused by HAdV-55 [14]. HAdVs can cause outbreaks, which usually occur in crowded places, such as hospitals, nursing homes, military bases, schools, and swimming pools, and these have been reported in several countries [15–19]. HAdV-B (HADV-3, -7, -14, and -55) cause outbreaks of respiratory-related diseases [4, 16, 20, 21], whereas HAdV-D (HAdV-8, -19, -37, -53, and -54) cause outbreaks of epidemic keratoconjunctivitis [18, 22, 23].

HAdV-G is a novel species that has been typed and named by using whole genome sequencing and phylogenetics rather than by applying traditional serology. At present, the specific and frequently mutated hexon gene of HAdV has been widely used in the molecular diagnosis and genotyping of this virus. Owing to the frequent recombination of HAdVs, as exemplified by HAdV-85/89 in Japan, HAdV-D56 in France, and the HAdV-55 and CBJ113 strains in China [15, 24–28], whole-genome sequencing remains the gold standard for proper classification of HAdVs [28]. Among them, HAdV-55, reconstituted from HAdV-B11 and HAdV-B14, has repeatedly caused outbreaks in densely populated areas such as schools and the military in China [15, 16]. The purpose of this study was to evaluate the epidemiological, clinical, and molecular characteristics of HAdV infections occurring among hospitalized children with respiratory tract infections (RTIs) in Beijing Friendship Hospital in China from April 2018–March 2019. In addition, this work explored the relationship between HAdV infection and RTI symptoms to provide information for the control and prevention of HAdV infection in China.

## Materials and methods

### Patient specimens

The 1572 nasopharyngeal aspirate (NPA) samples used in this study were collected from hospitalized children (aged < 14 years) with RTIs at Beijing Friendship Hospital during the period from April 2018–March 2019. Informed consent was received from the parents or guardians of the children enrolled in the study. An RTI was defined as an illness that presented during the previous week with at least two of the following clinical manifestations: fever, cough, nasal obstruction, expectoration, sneeze and dyspnoea. Patients who were diagnosed with pneumonia by chest radiography were also included in the study, even if they did not show the clinical features described above [29]. The collected samples were stored in virus preservation solution (1640 medium with 2.5 mg/mL Bovine Serum Albumin, 25 µg/mL amphotericin B and 1% Penicillin–Streptomycin Solution), transported to the laboratory on ice, and stored at − 80 °C until further processing. The clinical data were collected and sorted out from the hospital database.

### Detection of HAdVs and other common respiratory viruses

For molecular detection, total viral nucleic acid was extracted from 200 µL of each clinical NPA specimen by using the QIAamp MinElute Kit (Qiagen, Germany) in accordance with the manufacturer’s instructions. HAdV detection was performed by using a quantitative real-time polymerase chain reaction (qPCR) assay targeting the highly conserved 132-bp region of the HAdV hexon gene, as previously decribed [30]. TaqMan Universal PCR Master Mix (Applied Biosystems, USA) was used to amplify HAdV hexon DNA with specific primers (Forward:5′-GCCCCAGTGGTCTTACATGCACATC-3′; Reverse: 5′-GCCACGGTGGGGTTTCTAAACTT-3′) and probe (5′-FAM-TGCACCAGACCCGGGCTCAGGTACTCCGA-3′-TAMRA); qPCR was performed using the Mx3005P qPCR System (Agilent Stratagene, USA). Samples with a cycle threshold (CT) of < 38 were retested with qPCR to confirm their classification as positive samples. The positive samples were quantified by applying a standard curve, as described previously [31].

### Detection of viral co-infection in HAdV-positive specimens

The HAdV-positive specimens were screened for simultaneous co-infection with 15 common respiratory viruses: influenza virus types A, B, and C (IFV A/B/C), parainfluenza virus types 1–4 (HPIV 1–4), human coronavirus HKU1/229E/OC43/NL63, human respiratory syncytial virus (HRSV), human rhinoviruses (HRV), human bocavirus (HBoV) and human metapneumovirus (HMPV). All viruses were detected by qPCR (Additional file 1: Table S1) [30, 32–42], RNA viruses were tested using AgPath-ID™ One-Step RT-PCR Kit (Ambion, USA), and DNA viruses were tested using TaqMan™ Gene Expression Master Mix (Thermo Fisher, USA) in accordance the corresponding manufacture’s protocols.

### HAdV genotyping

Nested PCR targeting the hypervariable region of the HAdV hexon gene was employed for genotyping as previously described [31]. The outer primers used were forward 5′-GCCACCTTCTTCCCCATGGC-3′ and reverse 5′-GTAGCGTTGCCGGCCGAGAA-3′, and the internal primers were forward 5′-TTCCCCATGGCCCACAACAC-3′ and reverse 5′-GCCTCGATGACGCCGCGGTG-3′. Specimens that failed to be amplified were classified as untyped. Nested-PCR products were confirmed by sequencing, and a phylogenetic tree was constructed by applying the Maximum Likelihood (ML) method with MEGA 7.0 using 1000 bootstrap replicates. Reference HAdV strains (Additional file 1: Table S2) were selected based on the HAdV reference strain recommended by International Committee on Taxonomy of Viruses (ICTV) and also included CBJ113 strain (KR699642). Homology between sequences on the same evolutionary branch with CBJ113 was analyzed using BioEdit.

### Statistical analysis

Data analysis was performed using SAS 9.4 software, and the significance of the difference in rates among categorical data was tested by chi-squared and Fisher’s exact tests. Wilcoxon’s test and independent-samples *t-*test were used to analyze continuous variables. Two-sided *P*-values < 0.05 were considered indicative of statistical significance.

## Results

### HAdV epidemiology

**Fig. 1:**
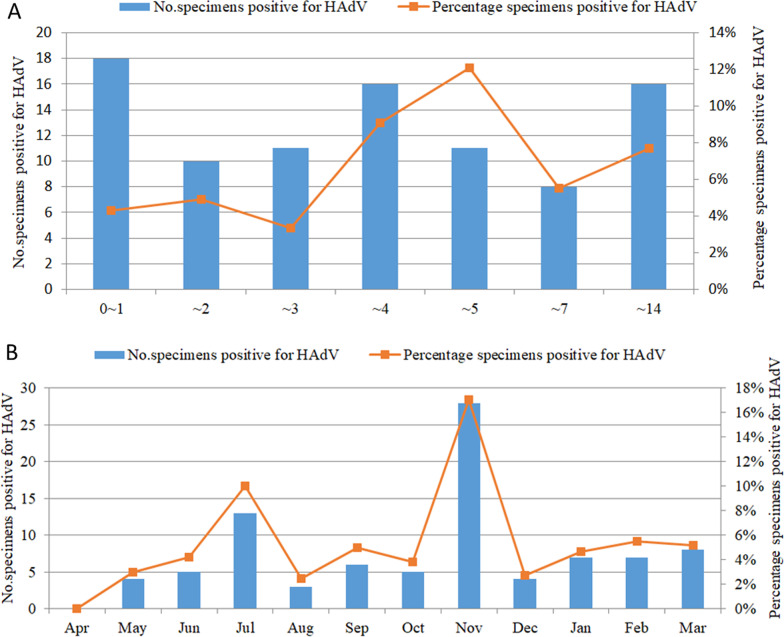
Age distribution (**a**) and seasonal distribution (**b**) of hospitalized children with RTIs who were infected with HAdV

A total of 1572 hospitalized children were enrolled during the period from April 2018–March 2019, among which 900 (57.25%) were male and 672 (42.75%) were female (sex ratio: 1.34:1). The age range was from 1 day old to 14 years old with a median age of 3 years old (IQR: 1–5 years old). Among the 1572 samples, 90 (5.72%) tested positive for HAdV. As shown in Table 1, among the 90 HAdV-positive children, 60 (66.67%) were male and 30 (33.33%) were female (sex ratio: 2:1). No significant difference in HAdV-positivity was observed between the male and female patients (*P* = 0.063). Infected children were aged range from 1 month to 13 years old (Fig. 1). The detection rate of HAdV infection differed significantly among age groups (*P* = 0.008); the HAdV detection rate was highest in the group aged > 4 but ≤ 5 years old (12.22%, 11/90). Cases of HAdV infection were detected throughout the whole year, but the month with the highest detection rate was November 2018 (17.07%, 28/164), followed by July 2018 (10%, 13/130). The HAdV detection rates differed significantly between spring, summer, autumn and winter (3.31%, 5.72%, 9.38% and 4.23%, respectively, *P* = 0.001).

**Table 1:** Age and sex differences among hospitalized children with HAdV infection

Variable	Number of children	Number of children positive for HAdV	Percentage children positive for HAdV (%)	*P* value
Age (years)				
0~1	420	18	4.29	0.008
~2	204	10	4.90	
~3	328	11	3.35	
~4	176	16	9.09	
~5	91	11	12.09	
~7	145	8	5.52	
~14	208	16	7.69	
Gender				
Male	900	60	6.67	0.063
Female	672	30	3.09	
Total	1572	90	5.72

### Detection of viral co-infection in HAdV-positive specimens

Among the 90 HAdV-positive specimens, single infection samples accounted for 38.89% (35/90), among these patients (23 males and 12 females, male–female ratio of 1.92:1), the difference between the numbers of male and female patients was not statistically different (*P* = 0.878). Of the 55 HAdV-positive children who were co-infected with other respiratory viruses, the most common co-infecting viruses were HRSV and HRV (Table 2). The viral load of the 90 HAdV positive samples ranged from 17 to 12.8 × 10^6^ copies/mL NPA. The log numbers of HAdV genome copies were 2.85 ± 1.45 and 2.63 ± 1.32 in the NPAs of children infected with HAdV only and those co-infected with HAdV and other respiratory virus, respectively; however there is no statistical difference in the viral load between HAdV mono- and co-infections (*P* = 0.061).

**Table 2:** HAdV coinfection with other respiratory viruses

Coinfection	Virus composition	Number of cases (%)
2 Viruses (n = 37)	HAdV + HRSV	19 (34.5)
	HAdV + HRV	6 (10.9)
	HAdV + IFV	6 (10.9)
	HAdV + HPIV	3 (5.4)
	HAdV + HBoV	1 (1.8)
	HAdV + HMPV	1 (1.8)
	HAdV + HCoV	1 (1.8)
3 Viruses (n = 14)	HAdV + HRSV + HRV	4 (7.3)
	HAdV + HRSV + IFV	3 (5.4)
	HAdV + IFV + HMPV	1 (1.8)
	HAdV + HRV + HMPV	1 (1.8)
	HAdV + HBoV + HPIV	1 (1.8)
	HAdV + HBoV + HMPV	1 (1.8)
	HAdV + HRSV + HMPV	1 (1.8)
	HAdV + HPIV + HRV	1 (1.8)
	HAdV + HPIV + HMPV	1 (1.8)
4 Viruses (n = 4)	HAdV + HPIV + HRV + HMPV	1 (1.8)
	HAdV + HCoV + HPIV + HRV	1 (1.8)
	HAdV + HRV + HBoV + HMPV	1 (1.8)
	HAdV + IFV + HBoV + HMPV	1 (1.8)

*IFV* influenza virus A–C; *HRV* human rhinovirus, *HBoV* human bocavirus, *HRSV* human respiratory syncytial virus, *HPIV* human parainfluenza virus 1–4, *HMPV* human metapneumovirus, *HCoV* human coronavirus (HKU1/229E/OC43/NL63)

### Clinical characteristics of HAdV infections

Among the 90 HAdV-positive children, the main diagnosis was bronchopneumonia (68.89%, 62/90), followed by mycoplasma pneumoniae pneumonia (8.89%, 8/90); only 3 cases were diagnosed with adenovirus pneumonia. The average duration hospitalization among these patients was 5.85 days. Eighty (88.89%) of the 90 children had an abnormal chest radiograph, and 48 (53.33%) of the 90 exhibited an elevated WBC count (> 10 × 10^9^ cells/L). The main clinical features of the HAdV infections included cough (83.33%, 75/90) and fever (temperature ≥ 38 °C; 90%, 81/90). Five cases experienced convulsion as a symptom. A small number of children presented with gastrointestinal symptoms, such as vomiting (14.44%, 13/90) and diarrhoea (4.44%, 4/90). There were no significant differences in the clinical characteristics of HAdV infection between HAdV-positive patients with or without a viral co-infection (Table 3). Among children with an exclusive HAdV infection (no viral co-infection), the relationships between viral load and patient age, sex, disease duration, and body temperature were not statistically significant (Table 4), but children with a high viral load were more likely to have a high fever (*P* = 0.041) and an elevated WBC count(*P* = 0.000).

**Table 3:** Clinical characteristics of children infected with HAdVs

Clinical characteristics	Single infection NO (%) (n = 35)	Co⁃infection NO (%) (n = 55)	Total No (%) (n = 90)	*P*
Fever (≥ 39℃)	28 (80.00)	38 (69.09)	66 (73.33)	0.254^a^
Abnormal chest radiograph	31 (88.57)	49 (89.09)	80 (88.89)	1.000^b^
WBC > 10	16 (45.71)	32 (58.18)	48 (53.33)	0.248^a^
Hospitalization > 7d	4 (11.43)	12 (21.82)	16 (17.78)	0.209^a^
Chill	10 (28.57)	10 (18.18)	20 (22.22)	0.248^a^
Shiver	4 (11.43)	5 (9.09)	9 (10.00)	0.731^b^
Cough	27 (77.14)	48 (87.27)	75 (83.33)	0.208^a^
Expectoration	26 (74.28)	43 (78.18)	69 (76.67)	0.670^a^
Nasal obstruction	10 (28.57)	17 (30.91)	27 (30.00)	0.814^a^
Rhinorrhea	13 (37.14)	29 (52.73)	42 (46.67)	0.149^a^
Sneeze	5 (14.29)	9 (16.36)	14 (15.56)	0.791^a^
Vomit	8 (22.86)	5 (9.09)	13 (14.44)	0.070^a^
Diarrhea	1 (2.86)	3 (5.45)	4 (4.44)	0.953^b^
Convulsions	3 (8.57)	2 (3.64)	5 (5.56)	0.600^b^
Dyspnea	0 (0)	1 (1.82)	1 (1.11)	1.000^c^

^a^Chi-squared test; ^b^continuity correction chi-squared test; ^c^Fisher’s exact test *P < 0.05

**Table 4:** Viral load and clinical characteristics of children infected with HAdVs

Clinical data		NO (%) (n = 35)	Mean viral load (log)	*P*
Age (months)	≤ 48	16	3.18 ± 1.78	0.312^a^
	> 48	19	2.56 ± 1.07	
Gender	Male	23	2.96 ± 1.58	0.903^a^
	Female	12	2.65 ± 1.20	
Hospitalization (d)	≤ 5	24	2.72 ± 1.28	0.364^a^
	> 5	11	3.18 ± 1.66	
Temperature (℃)	< 40	22	3.44 ± 1.40	*0.041^a^
	≥ 40	13	2.49 ± 1.39	
WBC (× 10^9^cells/L)	≤ 10	19	6.94 ± 1.48	*0.000^b^
	> 10	16	3.14 ± 1.70	

^a^Wilcoxon's rank sum test; ^b^independent-samples *t*-test; *P < 0.05

### Phylogenetic analysis of HAdVs

**Fig. 2:**
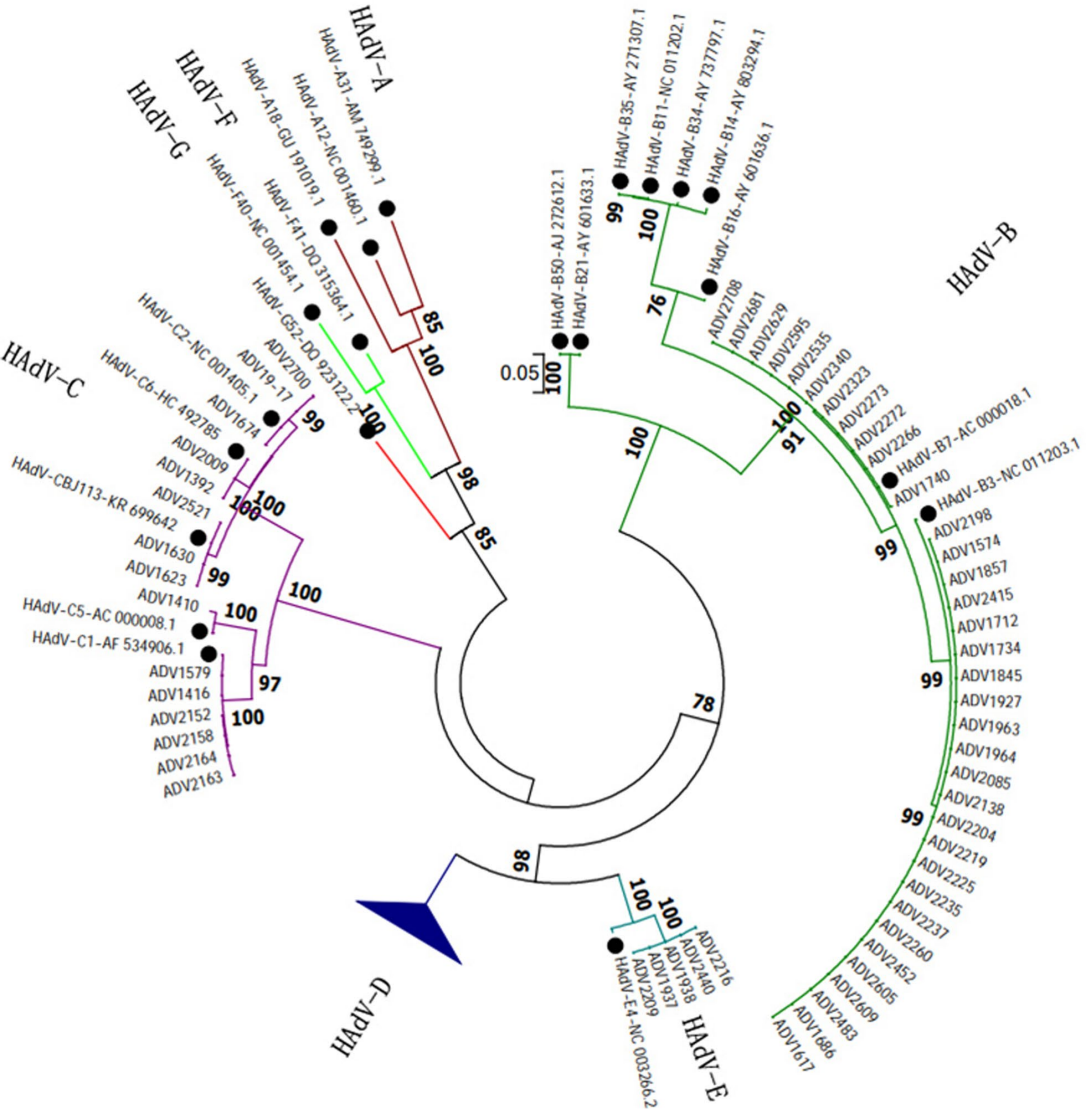
Phylogenetic analysis of HAdV hexon gene (758-bp) compared with reference strains. The reference strain is marked with “●”

The hexon gene (758-bp) was used for nested PCR amplification, and 55 HAdV-positive samples were amplified. Phylogenetic analysis revealed that 63.64% (35/55) of strains belonged to HAdV-B, including HAdV-B3 (68.57%, 24/35) and HAdV-B7 (31.43%, 11/35). Additionally, 27.27% (15/55) of the strains belonged to HAdV-C, including HAdV-C1 (40%, 6/15), HAdV-C2 (20%, 3/15), HAdV-C6 (13.33%, 2/15), HAdV-C5 (6.67%, 1/15) and CBJ113 (20%, 3/15). Furthermore, 5 cases were revealed to be HAdV-E, all of which were HAdV-E4 (Fig. 2). The genotyping of ADV1623/1630/2521 was consistent with that of CBJ113, and the homology between their hexon genes was 99.7%–99.8%.

## Discussion

The respiratory tract-related clinical symptoms caused by HAdV infections are similar to those caused by infection with IFA, HRSV and other respiratory pathogens. Consequently, correct diagnosis of HAdV infection is often difficult. In this study, qPCR and Sanger sequencing were used to analyse the phylogenetic sequence of the hexon gene, and the epidemiological characteristics and genotypic diversity of HAdVs in children hospitalized during the period from April 2018–March 2019 in Beijing, China were investigated. Of the 1572 collected specimens, 90 (5.73%) were positive for HAdV; this HAdV detection rate is very similar to that of the previous year in this hospital (5.64%) [31] and is also consistent with those reported from China and other countries (3.71%–35.5%) [31, 38–42]. The HAdV detection rate was 3.71% in Hebei Province, China. However, it was slightly higher among hospitalized children with RTIs in southern China; the HAdV detection rate in hospitalized children with RTIs during the period from 2009 to 2012 in Chongqing was 8.55%, and that in Hunan Province was 9.4%. It should be noted that different HAdV detection rates may be the result of differences in detection method, sample collection site, collection time and other factors. Thus, it is necessary to establish a unified and continuous epidemiological surveillance over a wider area.

The HAdV detection rate was not significantly affected by patient sex, but it was significantly affected by patients age (*P* = 0.008). The main age group affected by HAdV was children aged ≤ 5 years (73.3%, 66/90), specifically, the group of patients aged 3–5 years had the highest HAdV detection rate (10.11%, 27/267), whereas the group aged 2–3 years had the lowest (3.35%, 11/328). The reason for this difference remains to be determined.

Previous studies have shown that the HAdV detection rate is positively correlated with monthly average temperature, sunshine hours, and air temperature [39]. The number of HAdV infections in southern China reaches its peak during summer. In this study, the HAdV detection rate in Beijing had an obvious seasonal distribution difference (*P* = 0.001), and peaking in autumn (9.33%), which is consistent with a previous report by Duan et al. [43].

The clinical symptoms caused by HAdV infection in our patients were similar to those commonly caused by infections with other respiratory viruses, such as HRSV and IFV; their most common clinical symptoms and signs were fever and cough, and a few cases of HAdV also experienced gastrointestinal symptoms, such as vomit and nasal obstruction. The most common diagnosis among our HAdV-positive subjects was bronchopneumonia (68.89%, 62/90). The duration of hospital stay was generally less than 7 days, which is consistent with the results of previous studies. The co-infection of HAdVs and other respiratory viruses has been reported many times [39, 40]. In this study, the co-infection rate was 61.11% (55/90), and the viruses with the highest frequency of mixed infection were HRSV and HRV. No significant difference was observed in clinical symptoms and duration of hospitalization between the mono- and co-infections. The severity of HAdV infection is affected by many factors, including the patient age, immune status, and socioeconomic status. Although, some studies have shown that HAdV-7 may cause a more severe infection [44]; others have found that the HAdV type has no obvious influence on the severity of respiratory tract infection in children. Additionally, HAdV-infected patients with a long-lasting fever often experience more serious disease [40]. In agreement with previous work, this study found that there was no significant association between the HAdV genotype and disease severity (Additional file 1: Table S3), and only one child who was co-infected with HRSV had dyspnea. In the analysis of HAdV simple infection, the viral load in NPAs had no significant statistical association with patient age, sex or hospital stay duration, but children with a high viral load were more likely to have a high fever and elevated WBC count.

The most common species of HAdV worldwide, affecting the respiratory tracts of both adults and children, are HAdV-B, HAdV-C and HAdV-E, among which HAdV-B3 and HAdV-B7 are the main epidemic strains [44–46]. HAdV-55, which often causes outbreaks, is also detected at high rates in cases of adult respiratory tract infection [4, 6]. The prevalent HAdVs in China are mainly genotypes HAdV-2, -3 and -7. The dominant genotypes in northern China are HAdV-3 and -7, while those in southern China were HAdV-2 and 3 [43]. In this study, hexon gene sequencing and phylogenetic analysis were performed on 55 samples. The results show that HAdV species B and C were the most common species, accounting for 63.64% (35/55) and 29.10% (16/55) of HAdV cases, respectively. HAdV-B3 was the most common genotype (43.64%, 24/55), followed by HAdV-B7 (20.00%, 11/55), HAdV-C1 (10.91%, 6/55), and HAdV-E4 (9.09%, 9/55), which is consistent with other reports. HAdVs are prone to gene mutation and recombination [7, 25, 45, 47], and the CBJ113 strain isolated in Beijing in 2016 contained HAdV-C2, HAdV-C6, HAdV-C1, HAdV-C5 and HAdV-C57 sequences, which were recombined in several genes, including the hexon and fiber genes. Notably, three of the hexon sequences detected here were on the same branch as strain CBJ113, and they showed maximum homology with strain CBJ113. This study demonstrates that there are at least eight different HAdV genotypes circulating in Beijing; and that the HAdV species C strain CBJ113 has been prevalent in China for a long time. The hexon gene is commonly used for typing and is common in many molecular epidemiological studies of HAdV [48, 49]. However, because adenoviruses are prone to mutating and recombining, it is more accurate to use the whole genotype, which is a limitation of this study.

Our data allows CDC and health officials to understand the importance of adenovirus infection more deeply, thereby the government will give more attention and financial support to adenovirus research. In addition, our study can also provide clinicians more information of adenovirus infection, and patients can get the accurate diagnosis and better treatment for viral infection.

## Conclusion

This study described the epidemiological, clinical, and molecular characteristics of HAdV infections occurring among children with RTIs in a Chinese tertiary hospital during the period April 2017–March 2018. Our results show the latest trends of HAdV epidemic genotypes in Beijing, China. Notably, the HAdV-C strain CBJ113 has formed an epidemic trend in Beijing; therefore it is necessary to establish a nationwide epidemiological surveillance program for adenovirus infection because the epidemic data from a single region are not necessarily representative. The detection of HAdV should be carried out across multiple regions in China. Additionally, it is not sufficient to use only a single gene fragment for HAdV typing; the whole HAdV genome should be sequenced to provide a more comprehensive understanding of the evolutionary characteristics of HAdVs and to provide theoretical support for HAdV prevention and control strategies.

## Supplementary Information

**Additional file 1.** The tables of primers and probes used to detect co-infection respiratory viruses, reference strain of HAdV strains, clinical characteristics of children infected with different HAdV genotypes.

## Data Availability

Condensed anonymized data are available from the corresponding author on reasonable request.

## Abbreviations

CT: Cycle threshold
HAdV: Human adenoviruse
HBoV: Human bocavirus
HCoV: Human coronavirus
HMPV: Human metapneumovirus
HPIVs: Human parainfluenza viruses
HRV: Human rhinovirus
ICTV: International Committee on Taxonomy of Viruses
IFV-A: Influenza A
IQR: Interquartile Range
MEGA: Molecular evolutionary genetics analysis
ML: Maximum Likelihood method
NPAs: Nasopharyngeal aspirates
qPCR: Quantitative real-time polymerase chain reaction
RSV: Respiratory syncytial virus
RTIs: Respiratory tract infections
SAS: Statistics Analysis System
WBC: White Blood Count
