# Supplementary material for: Human adenoviruses in paediatric patients with respiratory tract infections in Beijing, China
Source: Virol J. 2021 Sep 23;18:191. doi: 10.1186/s12985-021-01661-6 (PMC8460180; doi:10.1186/s12985-021-01661-6)
Supplement: Supplementary file 1 — Additional file 1. The tables of primers and probes used to detect co-infection respiratory viruses, reference strain of HAdV strains, clinical characteristics of children infected with different HAdV genotypes. [file 12985_2021_1661_MOESM1_ESM.docx]

**Supplementary Table 1** Primers and probes used to detect co-infection respiratory viruses

| Primers | Primer sequence (5’-3’) | Reference |
| --- | --- | --- |
| IFV A Forward | GACCRATCCTGTCACCTCTGAC | [32] |
| IFV A Reverse | AGGGCATTYTGGACAAAKCGTCTA |  |
| IFV A Probe | FAM-TGCAGTCCTCGCTCACTGGGCACG-TAMRA |  |
| IFV B Forward | CCCACCRAGCAACAMGG | [33] |
| IFV B Reverse | CCTTCCGACATCAGCTTCACT |  |
| IFV B Probe | FAM-CCCGGAACCCATCCCCGGA-TAMRA |  |
| IFV C Forward | GGCAAGCGACATGCTGAAYA | [33] |
| IFV C Reverse | TCCAGCTGCYTTCATTTGCTTT |  |
| IFV C Probe | FAM-CTCTTCCTTCTGATTTTTTCAAA-TAMRA |  |
| HPIV 1 Forward | GTGATTTAAACCCGGTAATTTCTCA | [33] |
| HPIV 1 Reverse | CCTTGTTCCTGCAGCTATTACAGA |  |
| HPIV 1 Probe | FAM-ACCTATGACATCAACGAC-TAMRA |  |
| HPIV 2 Forward | ATGAAAACCATTTACCTAAGTGATGGA | [33] |
| HPIV 2 Reverse | CCTCCYGGTATRGCAGTGACTGAAC |  |
| HPIV 2 Probe | FAM-TCAATCGCAAAAGC-TAMRA |  |
| HPIV 3 Forward | CCAGGGATATAYTAYAAAGGCAAAA | [33] |
| HPIV 3 Reverse | CCGGGRCACCCAGTTGTG |  |
| HPIV 3 Probe | FAM-TGGRTGTTCAAGACCTCCATAYCCGAGAAA-TAMRA |  |
| HPIV 4 Forward | CAGAYAACATCAATCGCCTTACAAA | [34] |
| HPIV 4 Reverse | TGTACCTATGACTGCCCCAAARA |  |
| HPIV 4 Probe | FAM-CCMATCACAAGCTCAGAAATYCAAAGTCGT-TAMRA |  |
| HCoV-HKU1 Forward | AGTTCCCATTGCTTTCGGAGTA | [34] |
| HCoV-HKU1 Reverse | CCGGCTGTGTCTATACCAATATCC |  |
| HCoV-HKU1 Probe | FAM -CCCCTTCTGAAGCAA-TAMRA |  |
| HCoV-OC43 Forward | GCTCAGGAAGGTCTGCTCC | [34] |
| HCoV-OC43 Reverse | TCCTGCACTAGAGGCTCTGC |  |
| HCoV-OC43 Probe | FAM –TTCCAGATCTACTTCGCGCACATCC-TAMRA |  |
| HCoV-229E Forward | CGCAAGAATTCAGAACCAGAG | [34] |
| HCoV-229E Reverse | GGCAGTCAGGTTCTTCAACAA |  |
| HCoV-229E Probe | FAM–CCACACTTCAATCAAAAGCTCCCAAATG-TAMRA |  |
| HCoV-NL63 Forward | AGGACCTTAAATTCAGACAACGTTCT | [34] |
| HCoV-NL63 Reverse | GATTACGTTTGCGATTACCAAGACT |  |
| HCoV-NL63 Probe | FAM-TAACAGTTTTAGCACCTTCCTTAGCAACCCAAACA-TAMRA |  |
| RSV Forward | GGCAAATATGGAAACATACGTGAA | [35] |
| RSV Reverse | TCTTTTTCTAGGACATTGTAYTGAACAG |  |
| RSV Probe | FAM-CTGTGTATGTGGAGCCTTCGTGAAGCT-TAMRA |  |
| HRV Forward | TGGACAGGGTGTGAAGAGC | [36] |
| HRV Reverse | CAAAGTAGTCGGTCCCATCC |  |
| HRV Probe | FAM-TCCTCCGGCCCCTGAATG-TAMRA |  |
| HBoV Forward | CTGCTGCACTTCCTGATTCAAT | [37] |
| HBoV Reverse | GGAGCTTCTTCCAGAGATGTTC |  |
| HBoV Probe | FAM-ACTGCATCCGGTCTC-TAMRA |  |
| HMPV Forward | CATATAAGCATGCTATATTAAAAGAGTCTC | [38] |
| HMPV Reverse | CCTATTTCTGCAGCATATTTGTAATCAG |  |
| HMPV Probe | FAM-TGYAATGATGAGGGTGTCACTGCGGTTG-TAMRA |  |

**Supplementary Table 2** Reference strain of HAdV strains

| Accession# | Species | Genotype |
| --- | --- | --- |
| NC_001460.1 | HAdV-A | 12 |
| GU_191019.1 | HAdV-A | 18 |
| AM_749299.1 | HAdV-A | 31 |
| NC_011203.1 | HAdV-B | 3 |
| AC_000018.1 | HAdV-B | 7 |
| NC_011202.1 | HAdV-B | 11 |
| AY_803294.1 | HAdV-B | 14 |
| AY_601636.1 | HAdV-B | 16 |
| AY_601633.1 | HAdV-B | 21 |
| AY_737797.1 | HAdV-B | 34 |
| AY_271307.1 | HAdV-B | 35 |
| AJ_272612.1 | HAdV-B | 50 |
| AF_534906.1 | HAdV-C | 1 |
| NC_001405.1 | HAdV-C | 2 |
| AC_000008.1 | HAdV-C | 5 |
| HC_492785 | HAdV-C | 6 |
| AB_448767.1 | HAdV-D | 8 |
| NC_010956.1 | HAdV-D | 9 |
| DQ_149615.1 | HAdV-D | 10 |
| DQ_149616.1 | HAdV-D | 13 |
| AB_562586.1 | HAdV-D | 15 |
| EF_121005.1 | HAdV-D | 19 |
| DQ_149619.1 | HAdV-D | 20 |
| FJ_404771.1 | HAdV-D | 22 |
| DQ_149621.1 | HAdV-D | 23 |
| DQ_149622.1 | HAdV-D | 24 |
| DQ_149623.1 | HAdV-D | 25 |
| EF_153474.1 | HAdV-D | 26 |
| DQ_149625.1 | HAdV-D | 27 |
| FJ_824826.1 | HAdV-D | 28 |
| AB_562587.1 | HAdV-D | 29 |
| DQ_149628.1 | HAdV-D | 30 |
| DQ_149629.1 | HAdV-D | 32 |
| DQ_149630.1 | HAdV-D | 33 |
| GQ_384080.1 | HAdV-D | 36 |
| DQ_900900.1 | HAdV-D | 37 |
| DQ_149633.1 | HAdV-D | 38 |
| DQ_149634.1 | HAdV-D | 39 |
| DQ_149635.1 | HAdV-D | 42 |
| DQ_149636.1 | HAdV-D | 43 |
| DQ_149637.1 | HAdV-D | 44 |
| DQ_149638.1 | HAdV-D | 45 |
| AY_875648.1 | HAdV-D | 46 |
| DQ_149640.1 | HAdV-D | 47 |
| EF_153473.1 | HAdV-D | 48 |
| DQ_393829.1 | HAdV-D | 49 |
| DQ_149642.1 | HAdV-D | 51 |
| FJ_169625.1 | HAdV-D | 53 |
| AB_333801.2 | HAdV-D | 54 |
| NC_003266.2 | HAdV-E | 4 |
| NC_001454.1 | HAdV-F | 40 |
| DQ_923122.2 | HAdV-G | 52 |
| KR_699642 | HAdV-C | CBJ113 |

**Supplementary Table 3** Clinical characteristics of children infected with different HAdV genotypes

| Species | HAdV-B(35) | HAdV-C(15) | HAdV-E(5) | *P* |
| --- | --- | --- | --- | --- |
| Single infection | 19 | 6 | 1 | 0.269^a^ |
| Co⁃infection | 16 | 9 | 4 |  |
| Viral load（log） | 2.86±1.52 | 2.8±1.52 | 3.4±1.14 | 0.499^b^ |
| Hospitalization | 5.28±1.92 | 6.87±3.94 | 5.8±2.05 | 0.454 ^b^ |
| Fever (≥39℃) | 10 | 2 | 2 | 0.366 ^a^ |

a, Continuity correction chi-squared test; b, Wilcoxon's rank sum test
